# Prediction of Post-operative Long-Term Outcome of the Motor Function by Multimodal Intraoperative Neuromonitoring With Transcranial Motor-Evoked Potential and Spinal Cord-Evoked Potential After Microsurgical Resection for Spinal Cord Tumors

**DOI:** 10.3389/fsurg.2022.883832

**Published:** 2022-05-04

**Authors:** Shinsuke Yamada, Satoshi Kawajiri, Hidetaka Arishma, Makoto Isozaki, Takahiro Yamauchi, Ayumi Akazawa, Masamune Kidoguchi, Toshiaki Kodera, Yoshinori Shibaike, Hideto Umeda, Yu Tsukinowa, Ryota Hagihara, Kenichiro Kikuta

**Affiliations:** Department of Neurosurgery, Division of Medicine, Faculty of Medical Sciences, University of Fukui, Fukui, Japan

**Keywords:** spinal tumor, motor-evoked potential, motor function, extremity, long-term outcome, McCormick score

## Abstract

**Objective:**

To examine the effect of multimodal intraoperative neuromonitoring on the long-term outcome of motor function after microsurgical resection for spinal cord tumors.

**Materials and Methods:**

Consecutive fourteen patients with spinal tumors who were surgically treated at the University of Fukui Hospital between 2009 and 2020 [M:F = 10:4, ages ranging from 22 to 83 years (mean ± SD = 58 ± 21 years)] were included in this study. There were eight intra-axial tumors and six extra-axial tumors. There were four patients with hypertension, two patients with diabetes mellitus, and four patients with hyperlipidemia. Three patients were under antithrombotic medication, two were under steroid medication, four were current smokers, and four were current drinkers. Manual muscle test (MMT) of the upper and lower extremities of the patients was examined before surgery, 2 weeks after surgery, and at the final follow-up. The mean follow-up period was 38 ± 37 months. McCormick scores were examined before surgery and at the final follow-up. Microsurgical resection of the tumor was underwent through the posterior approach under transcranial motor-evoked potential (TcMEP) monitoring. The MEP of 46 extremities was recorded during the surgery. Gross total resection was achieved in 13 of 14 surgeries. Spinal cord-evoked potential (Sp-SCEP) monitoring was performed in eight of 14 patients.

**Results:**

The length of peritumoral edema was significantly longer in patients with deterioration of McCormick scores than in patients with preservation of McCormick scores (*p* = 0.0274). Sp-SCEP could not predict the deterioration. The ratio of MEP at the beginning of the surgery to that at the end of the surgery was the only significant negative factor that predicts deterioration of motor function of the extremity at the final follow-up (*p* = 0.0374, odds ratio [OR] 1.02E-05, 95% CI 9.13E+01–7.15E+18). A receiver operating characteristic (ROC) analysis revealed that the cutoff value of the ratio of MEP to predict the deterioration at the final follow-up was 0.23 (specificity 100%, specificity 88%, positive predictive value 100%, and negative predictive value 88%) to predict deterioration at the final follow-up.

**Conclusions:**

Ratio MEP was the most significant negative factor to predict the deterioration of motor weakness at spinal tumor surgery. The setting of the cutoff value should be more strict as compared to the brain surgery and might be different depending on the institutions.

## Introduction

Surgery of spinal tumors, especially the intramedullary spinal cord tumor (IMSCT), has potential risk of sensory-motor dysfunction after surgery. Although Elsberg reported the first case of total resection of IMSCT in 1916 (1), most cases were still treated by radiation therapy after biopsy because of the high morbidity and mortality rates (2). Technical innovation, such as microscope and bipolar coagulator, has reduced the morbidity and mortality of total resection of SCT (3). Intraoperative neuromonitoring (IONM) by sensory-evoked potential (SEP) was first indicated in scoliosis surgery, which also had the risk of postoperative motor dysfunction (4). SEP could detect relatively severe injury, such as transverse spinal cord injury, but could not detect partial injury, such as anterior spinal artery syndrome (5) or motor dysfunction after surgery, for intramedullary tumor (6).

Intraoperative neuromonitoring with motor-evoked potential (MEP) by transcranial stimulation (TcMEP) in spinal surgery was first indicated in surgery of cervical spine (7, 8) and surgery of spinal cord injury (9). Afterward, MEP was applied to spinal tumor surgery (10, 11). Surgery of IMSCT under MEP was significantly related to the good outcomes of adult patients (10) and disappearance of MEP was significantly related to the deterioration of motor function immediately after surgery and at the final follow-up (11). MEP also could significantly increase the gross total resection rate (12). In surgery for 500 IMSCTs, complete resection could be achieved in 77.2% of defined endophytic tumors and 41.7% of diffuse tumors by the implementation of IONM with MEP (13). Preoperative motor dysfunction and intraoperative worsening of MEP were significantly related to the surgical outcomes (14).

The sensitivity and specificity of MEP in spinal surgery were 83 and 86%, respectively, while those of brain surgery were 100 and 62% (15, 16). Analysis of the motor function of 150 muscles in surgery of 250 IMSCTs under TcMEP provided a 5.9% of the false-positive rate, 7% of false-negative rate, and 27% of record failure rate, while TcMEP was successfully recorded in 96% of 216 aneurysm surgeries (17). TcMEP seemed more unstable or less available in spinal surgery than in brain surgery.

D-wave, directly recorded MEP from the epidural electrode after transcranial stimulation, had also developed and used in combined with TcMEP. A recent report showed that TcMEP had the highest sensitivity and D-wave had the highest specificity among TcMEP, SEP, and D-wave 19 months after surgery of 28 IMSCTs (18).

Although MEP or D-wave was proved to be the most significant predictive factor of postoperative motor dysfunction (18), the cutoff value for the prediction of postoperative motor dysfunction remains unclear. Fifty percent reduction of MEP has been used as the warning value in aneurysm surgery (10, 15), the value seemed not enough for accurate prediction in surgery for a spinal tumor. Some investigators reported more strict cutoff values, such as 70 or 80%, that might be necessary to have MEP monitoring reliable in spinal surgery (12, 16, 17). In this study, we examined the effect of multimodal mIONM with TcMEP and spinal cord-evoked potential (Sp-SCEP) on the long-term outcomes of motor function after microsurgical resection for spinal cord tumors (SCTs) with logistic regression analysis of the factors that affect surgical outcomes and with receiver operating characteristic (ROC) analysis of the cutoff value of MEP for the prediction of postoperative motor dysfunction.

## Materials and Methods

### Patients

Consecutive fourteen patients with spinal who were surgically treated at the University of Fukui Hospital between 2009 and 2020 were enrolled in this retrospective study. There were ten men and four women, with ages ranging from 22 to 83 years (mean = 58 ±21 years). There were eight intra-axial tumors and six extra-axial tumors. The mean length of the lesion and mean length that of peritumoral edema were 2.5 ±1.2 vertebral bodies and 1.6 ±1.8 vertebral bodies, respectively. As for underlying diseases, there were four patients with hypertension, two patients with diabetes mellitus, and four patients with hyperlipidemia. Three patients were under antithrombotic medication, two were under steroid medication, four were current smokers, and four were current drinkers. Manual muscle test (MMT) of the upper and lower extremities of the patients was examined before surgery, 2 weeks after surgery, and at the final follow-up. McCormick scores of the patients were examined before surgery and at final follow-up. IONM with MEP underwent in all cases and the ratio of the amplitude of MEP at the end of surgery to that at the beginning of surgery in each extremity was calculated as Ratio MEP. Among them, the most decreased ratio in each patient was described as the worst ratio of MEP. IONM with Sp-SCEP was carried out in 8 patients. The ratio of amplitude at the end of surgery to that at the beginning of surgery was described as the ratio of Sp-SCEP. Gross total resection was achieved in 13 patients. Follow-up period was ranged from 1 to 106 months (mean ± SD = 38 ± 37 months; Table 1).

**Table 1:** Summary of clinical data for the 14 patients receiving spinal tumor surgery.

**Case**	**Age**	**Sex**	**Lesion**	**Site**	**Intraaxial or extraaxial**	**Length of lesion (vertebra)**	**Length of edema (vertebra)**	**Preopeartive McCormick scores**	**Tc-MEP**	**Worst ratio of MEP**	**Sp-SCEP**	**Ratio of Sp-SCEP**	**Gross total removal**	**Follw up period (months)**	**Postopeartive McCormick scores**
1	68	M	Ependymoma	C4-6	Intraaxial	2	5	3	+	0.45	−		+	1	4
2	36	M	Tumor with neurenteric cyst	C2	Intraaxial	3	1	3	+	0.08	+	0.33	−	68	3
3	36	M	Hemangioblastoma	T5-7	Extraaxial	4	4	1	+	0.59	+	1.0	+	17	1
4	79	F	Meningioma	T3-5	Extraaxial	1	0	3	+	0.6	−		+	18	1
5	45	M	Schwannnoma	C2	Extraaxial	1	0	2	+	1.0	−		+	30	1
6	62	F	Meningioma	T10	Extraaxial	1	0	1	+	0.3	+	0.8	+	50	1
7	75	M	Meningioma	T3-5	Extradural	2	0	4	+	1.29	+	0.6	+	3	4
8	31	M	Ependymoma	Th10-11	Intraaxial	2	1	2	+	0	+	1.0	+	72	3
9	72	M	Myxopapillary ependymoma	cauda equina	Intraaxial	1	1	2	+	0.09	−		+	59	3
10	28	F	Ependymoma	C4-T6	Intraaxial	13	2	4	+	0.23	+	1.0	+	1	4
11	65	M	Meningioma	C7-T1	Extraaxial	1	4	1	+	0.91	+	1.0	+	99	3
13	22	M	Schwannoma	C3	Extraaxial	1	0	1	+	0.56	−		+	2	1
12	83	M	Schwannoma	T5-7	Extraaxial	1	1	1	+	0	+	0.6	+	106	1
14	77	M	Ependymoma	L1	Intraaxial	1	4	2	+	0.77	−		+	1	3

### Surgical Resection

All patients received microsurgical resection of the tumor through a posterior approach under tcMEP monitoring. Corkscrew-shaped stimulatory electrodes of tcMEP (Unique Medical, Tokyo, Japan) were placed at the points of 1 cm posterior, 5 cm lateral to Cz. Train of five electrical stimulation with 120 mA, 500 Hz, 0.2 ms was performed. Electromyography of the thenar muscle of upper extremities and abductor pollicis muscle of lower extremities was recorded. The ratio of the amplitude of electromyography of each muscle at the end of the surgery to that at the beginning of the surgery was defined as the change of MEP. Total MEPs of 46 extremities were recorded during the surgery. The ratio of MEP at the beginning of the surgery to that at the end of the surgery was defined as Ratio MEP. Gross total resection was achieved in 13 of 14 surgeries. For Sp-SCEP, a bipolar catheter electrode was inserted into the epidural space at the rostral and the caudal side of laminectomy. Direct medullary electrical stimulations (duration: 0.1 ms, frequency 5 Hz, intensity: 1.5 mA) were performed by the bipolar electrode at the caudal side. SEP was detected by the electrode at the rostral side (high pass filter: 3,000 Hz, low pass filter: 10 Hz, 100 averaging; Table 1).

### Statistical Analysis

Univariate analysis was performed with Pearson's chi-squared test or Fisher's exact test for categorical variables or with the Mann-Whitney U-test for numeric variables. Forward and backward stepwise logistic regression analyses with the Akaike Information Criterion (AIC) to determine the associations of potential confounders for deterioration of motor weakness at MMT at the final follow-up with the Ratio MEP, presence of preoperative motor weakness, age and sex of the patient, length of the lesion, length of peritumoral edema, history of hypertension, diabetes mellitus, hyperlipidemia, presence of antithrombotic medication, steroid medication, smoking, and drinking. ROC analysis was performed to analyze the relationship between Ratio MEP and the deterioration of MMT of 46 extremities 2 weeks after surgery and at final follow-up.

All statistical analyses were performed using JMP 15.2.0 (SAS Institute, Cary, NC, USA) and R (R Foundation for Statistical Computing, Vienna, Austria), with an error probability of <0.05.

## Results

### Univariate Analysis of Factors Affecting the Deterioration of McCormick Scores at the Final Follow-Up

Postoperative deterioration of McCormick scores was observed in 5 patients (Table 1). There were 7 patients with a worst MEP ratio of less than 0.5 and 5 patients with worst MEP ratio of less than 0.3. However, the MEP ratio was not related to the postoperative deterioration of McCormick score. There was no difference in the age, sex, the number of patients with intra-axial tumor, gloss total resection, length of the lesion, the number of patients with hypertension, diabetes mellitus, antithrombotic medication, steroid medication, current smoking, and drinking between the patients with deterioration of McCormick scores and the patients with preservation of McCormick scores. Only the length of peritumoral edema was significantly longer in the patients with deterioration of McCormick scores than in the patients with preservation of McCormick scores (*p* = 0.0274; Table 2).

**Table 2:** Univariate analysis of factors associated with the deterioration of McCormick score at the final follow-up.

	**Postoperative deterioration of McCormick score**	**Postoperative preservation or improvement of McCormick score**	***p* value**
Number of patients	4 (28.6%)	10 (71.4%)	
Age (years old)	60.3 ± 20.2	56.5 ± 7.0	0.7773
Female	0 (0%)	4 (40%)	0.2507
Intraaxial tumor	3 (75%)	5 (50%)	0.5804
Length of lesion	0.82 ± 0.41	1.25 ± 0.40	0.2896
Length of edema	1.73 ± 0.87	1.29 ± 0.41	0.0274^*^
Gloss total resection	4 (100%)	8 (80%)	1.0000
Hypertension	0 (0%)	4 (0%)	0.2507
Diabetes mellitus	1 (25%)	1 (10%)	0.5055
Hyperlipidemia	0 (0%)	4 (40%)	0.2507
Antithrombic medication	1 (25%)	2 (20%)	1.0000
Steroid medication	0 (0%)	2 (20%)	1.0000
Smoking	1 (25%)	3 (30%)	1.0000
Drinking	2 (20%)	2 (20%)	0.5205

* *Means “the difference is statistically significant”*.

Of the 8 patients with surgery under Sp-SCEP monitoring, the ratio of Sp-SCEP was 0.33 in one patient, 0.6 in two patients, and 0.8 in one patient. However, there was no one with deterioration of McCormick scores in those patients. Among the remaining 4 patients without the reduction of Sp-SCEP, there were two (50%) patients with deterioration of McCormick scores.

### Logistic Regression Analysis of Factors Affecting the Deterioration of MMT of 46 Extremities at the Final Follow-Up

Forward and backward stepwise logistic regression analyses with AIC using 13 variates revealed that the prediction model that consisted of six factors of drinking, hyperlipidemia, hypertension, presence of preoperative motor weakness, Ratio MEP, and smoking gave the minimal AIC scores (21.4). Among those factors, Ratio MEP was the only significant negative factor that predicted deterioration of motor weakness at the final follow-up (*p* = 0.0374, odds ratio [OR] 1.02E-05, 95% CI 9.13E+01–7.15E+18; Table 3).

**Table 3:** Results of logistic regression analyses regarding the preservation of motor function at the final follow-up and the Akaike Information Criterion (AIC) values.

**Variable**	**Estimate**	***P* value**	**OR**	**95%CI**	
Drinking	−16.39	0.0639	7.613821E-08	2.008602E-21	4.453208E-02
Hyperlipidemia	−19.15	0.1267	4.85E-09	5.37E-22	7.00E-03
Hypertension	2.08	0.3306	8.032087E+00	1.11E-01	1.18E+03
Preoperative motor weakness at MMT	−11.62	0.062	8.97E-06	3.38E-13	4.20E-02
Change of MEP	−11.49	0.0374^*^	1.02E-05	9.210130E-12	2.94E-02
Smoking	17.33	0.0618	3.36E+07	9.127656E+01	7.15E+18
					AIC=24.7

* *Means “the difference is statistically significant”*.

### ROC Analysis Between the Change of MEP and the Deterioration of MMT of 46 Extremities 2 Weeks After Surgery and Final Follow-Up

**Figure 1:**
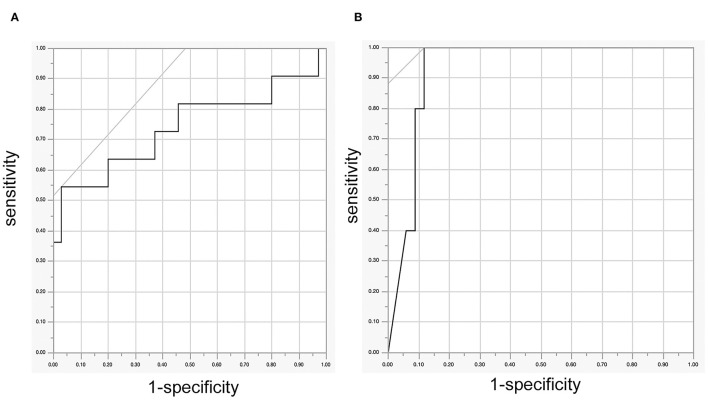
**(A)** ROC curve between the Ratio motor-evoked potential (MEP) and the deterioration of the motor function of 46 extremities 2 weeks after surgery, **(B)** ROC curve between Ratio MEP and the deterioration of the motor function of 46 extremities at the final follow-up. ROC, receiver operating characteristic.

Receiver operating characteristic analysis revealed that Ratio MEP was significantly related to the deterioration of the motor function of extremities 2 weeks after surgery (*p* = 0.0173, Figure 1A). The cutoff value of the Ratio MEP to predict motor function of extremities 2 weeks after surgery was 0.17 (specificity 55%, specificity 99.7%, positive predictive value 60%, and negative predictive value 97%). ROC analysis also revealed that Ratio MEP was significantly related to the deterioration of the motor function at the final follow-up (*p* = 0.0001, Figure 1B). The cutoff value of Ratio MEP to predict the motor function of extremities at the final follow-up was 0.23 (sensitivity 100%, specificity 88%, positive predictive value 100%, and negative predictive value 88%).

### Representative Cases

#### Case 1

**Figure 2:**
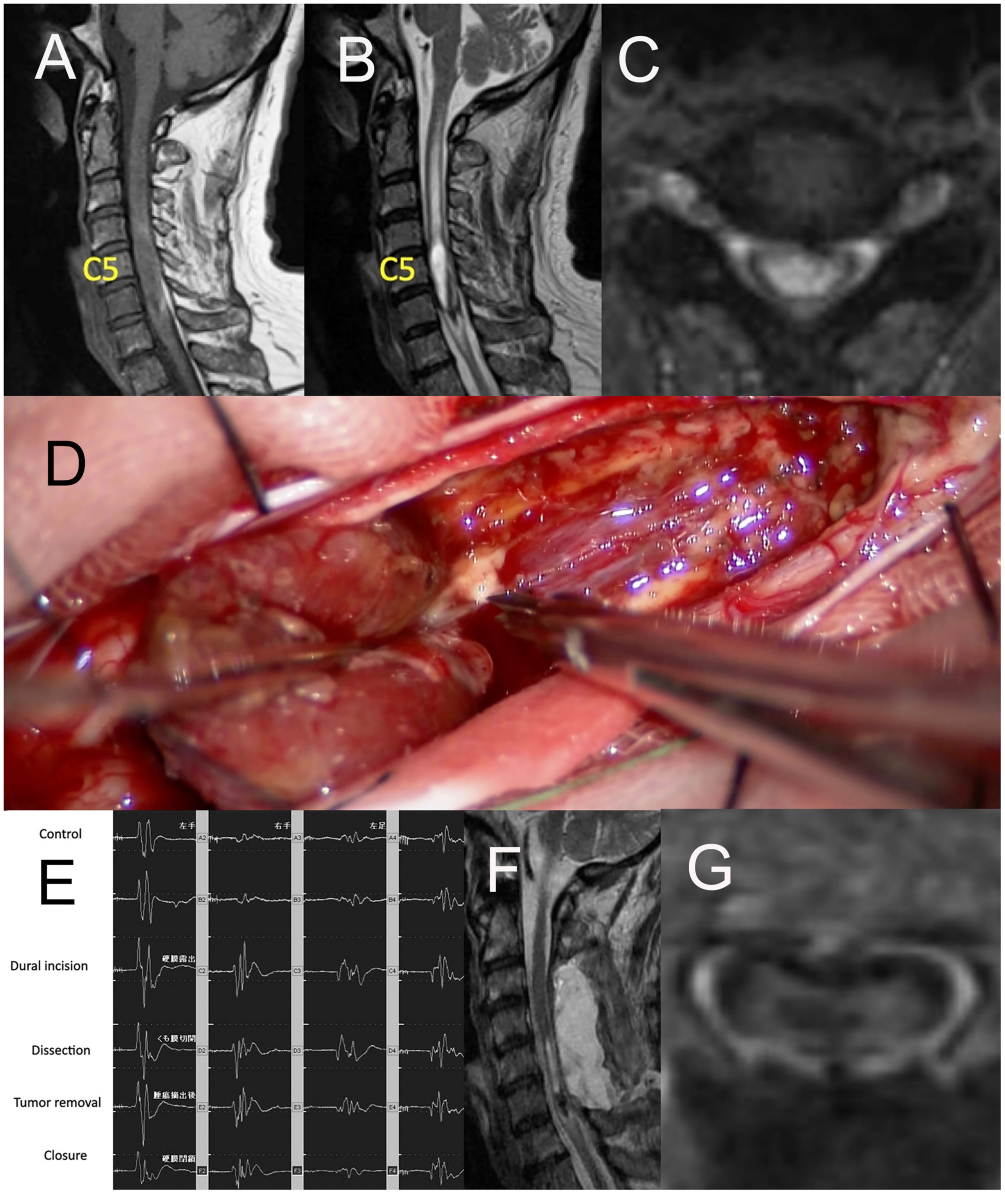
**(A)** Sagittal image of preoperative Gd-enhanced T1WI of MRI, **(B)** the axial image of preoperative T2WI of MRI, **(C)** the axial image of preoperative Gd-enhanced T1WI of MRI, **(D)** intraoperative photograph of tumor resection through midline myelotomy by posterior approach, **(E)** Ratio motor-evoked potential (MEP) of both UE and LE ranged from 91 to 108%, **(F)** Sagittal image of postoperative Gd-enhanced T1WI of MRI, **(G)** the axial image of postoperative Gd-enhanced T1WI of MRI. Gd, gadolinium; TIWI, T1-weighted image; MRI, magnetic resonance imaging; T2WI, T2-weighted image; UE, upper extremity; LE, lower extremity.

A 68-year-old man with ependymoma had been suffering from progressive paresthesia, fine movement disorder, reduction of grasping power of bilateral upper extremities, and gait disturbance for 2 months. Preoperative gadolinium (Gd)-enhanced T1-weighed image (T1WI) of magnetic resonance imaging (MRI) (Figures 2A,C) demonstrated intra-axial tumor of the cervical spine between C4 and C6. Preoperative T2-weighted image (T2WI) of MRI illustrated edema around the tumor ranging from C2 level and C6 level (Figure 2B). Gross total resection of the tumor was achieved through midline myelotomy by posterior approach (Figure 2D). The change in the amplitude of MEP of both upper and lower extremities (UE: upper extremity, LE: lower extremity) had ranged from 91 to 108% (Figure 2E). Postoperative Gd-enhanced T1WI of MRI (Figures 2F,G) showed gross total resection of the tumor.

#### Case 2

**Figure 3:**
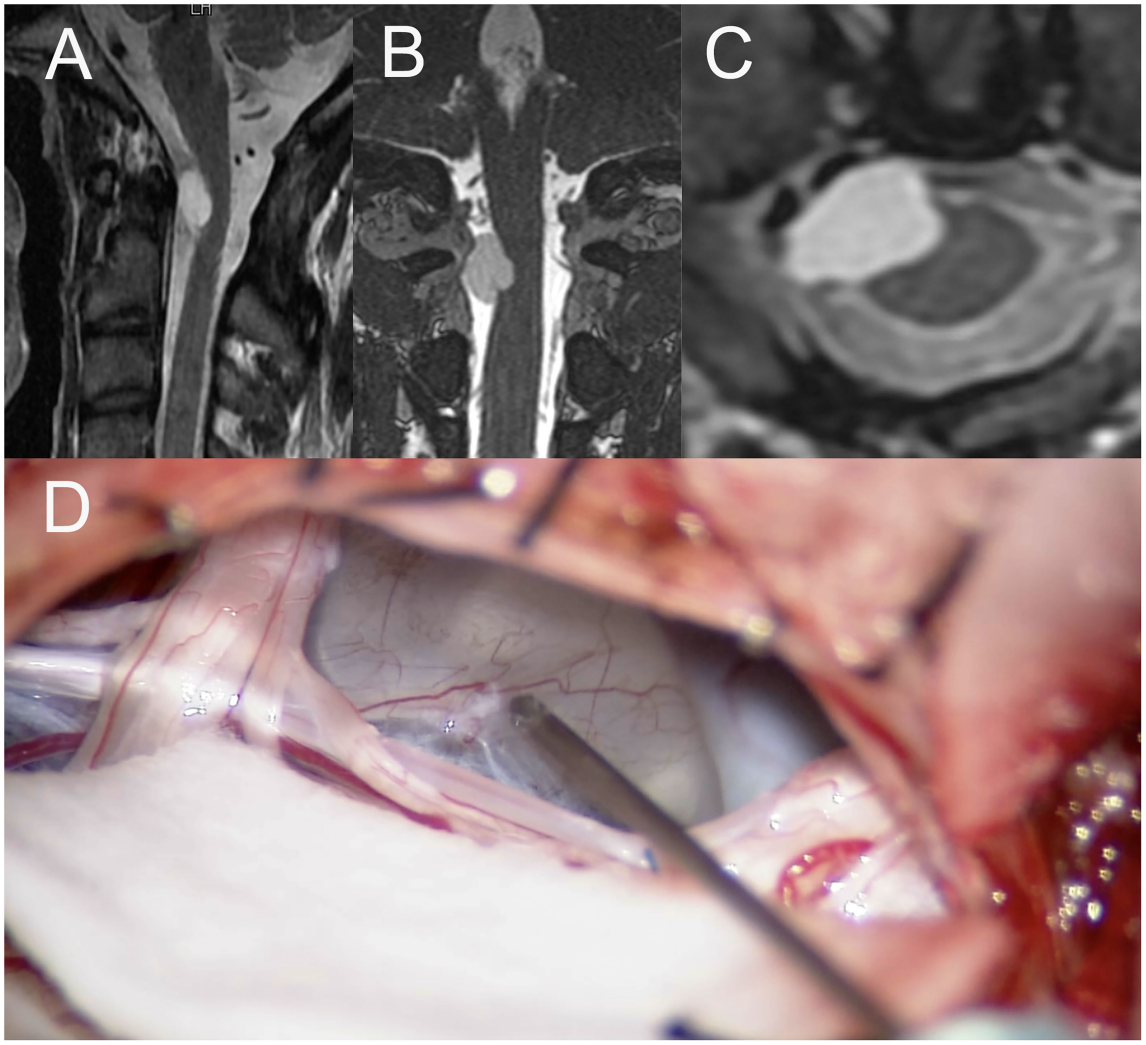
**(A)** Sagittal image of preoperative T1WI of MRI, **(B)** the coronal image of preoperative T1WI of MRI, **(C)** the axial image of preoperative Gd-enhanced T1WI of MRI, **(D)** intraoperative photograph of tumor resection by posterior approach. TIWI, T1-weighted image; MRI, magnetic resonance imaging; T2WI, T2-weighted image, Gd, gadolinium.

**Figure 4:**
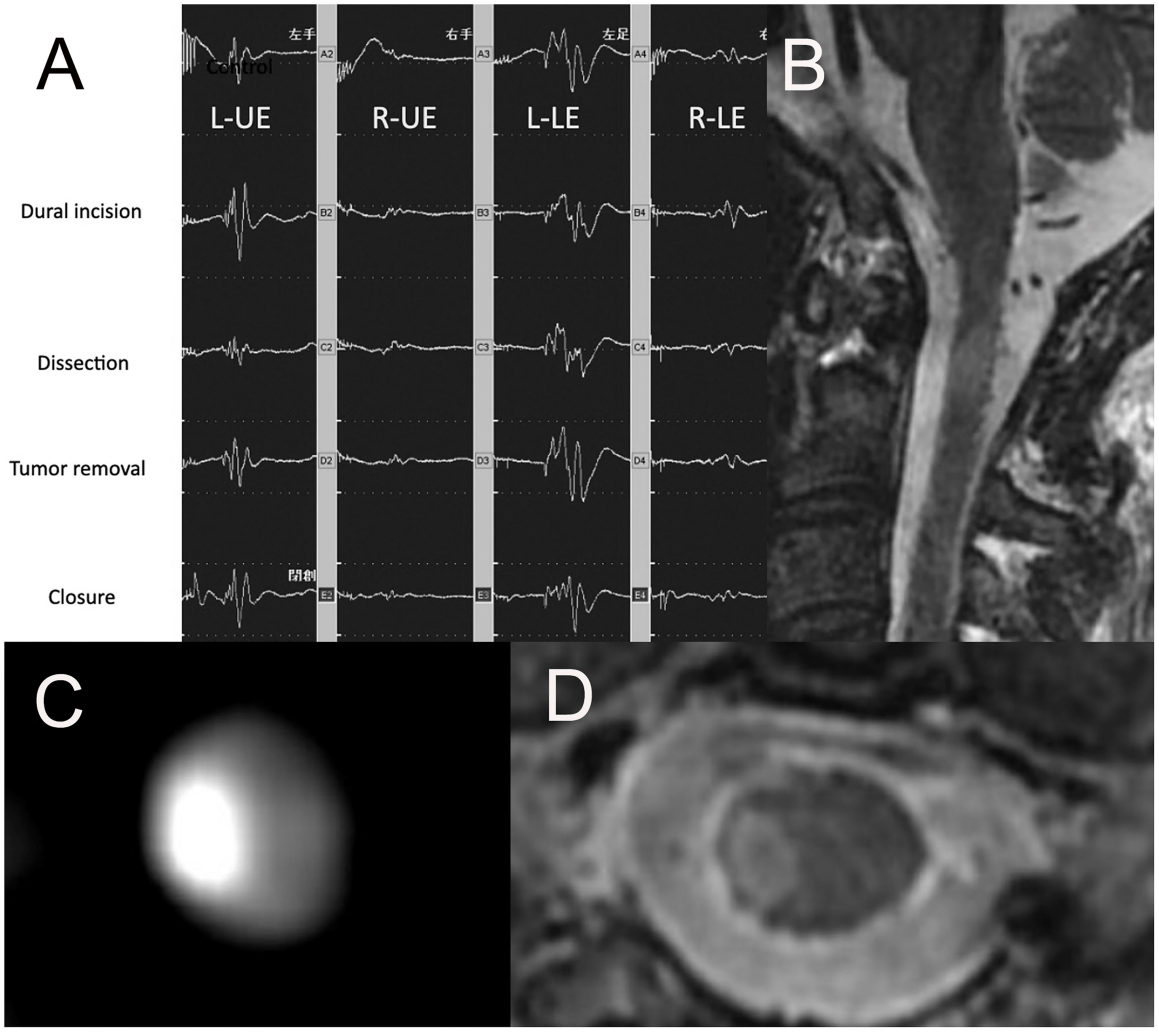
**(A)** Ratio motor-evoked potential (MEP) of right UE and right LE suddenly reached down to 0 and 30% compared to the initial value, **(B)** the sagittal image of postoperative T1WI of MRI, **(C)** the axial image of postoperative DWI of MRI, **(D)** the axial image of postoperative FLAIR of MRI. UE, upper extremity; LE, lower extremity; TIWI, T1-weighted image; MRI, magnetic resonance imaging; T2WI, T2-weighted image; DWI, diffusion-weighted imaging; FLAIR, fluid-attenuated inversion recovery.

A 36-year-old man with a tumor with a neurenteric cyst had been suffering from right occipital pain for 6 months. Preoperative T1WI of magnetic MRI (Figures 3A–C) demonstrated an intra-axial tumor of the cervical spine at C2 without edema around the tumor. Gross total resection of the tumor was achieved by a posterior approach (Figure 3D). However, during dissection of the tumor, the amplitude of MEP of the right upper and right lower extremities suddenly reached down to 0 and 30% as compared to the initial value, respectively (Figure 4A). Postoperative T1WI of MRI (Figure 4B) showed gross total resection of tumor. Postoperative diffusion-weighted image (DWI) of MRI (Figure 4C) indicated the occurrence of acute ischemic stroke of the right cervical spinal cord. Postoperative fluid-attenuated inversion recovery image (FLAIR) of MRI (Figure 4D) also showed the occurrence of stroke. The patient exhibited right hemiparesis, right hypesthesia of deep sensation, left hypesthesia of superficial sensation, increased deep tendon reflex, and right ankle clonus (Brown-Sequard syndrome) after surgery.

## Discussion

Since IONM with TcMEP was more unstable or less available in spinal surgery than in brain surgery, multimodal IONM (mIONM) was introduced. mIONM with TcMEP and SEP was first applied to spinal surgery (19–21). The sensitivity and specificity of the mIONM in surgery for spinal deformity or EMSCT or EDSCT were relatively high (20) and were significantly related to the good McCormick scores at the final follow-up in surgery of IMSCT (22). However, the sensitivity and specificity regarding the deterioration of McCormick scores were 73 and 78%, which were still low (23). In surgery for 127 cauda equina tumors, the sensitivity of root injury by triggered electromyogram (tEMG) monitoring was also 37.5%. IONM with tEMG, TcMEP, and SEP might improve the accuracy of the prediction (24).

Sensory-evoked potential induced by direct spinal cord stimulation (Sp-SCEP) had developed as an alternative option of SEP. Sp-SCEP was safe, stable, and reliable monitoring and was easily combined with TcMEP (25–27). D-wave was the potential directly recorded from the spinal cord by the epidural electrode induced by transcranial stimulation and had also developed as a more stable option for TcMEP. In surgery of 57 IMSCTs, mIONM with TcMEP, SEP, and D-wave was the significant predictive factor of postoperative motor dysfunction (area under the curve (AUC) 0.98) and D-wave was more significant than Tc-MEP or SEP (28).

While MEP or D-wave was proved to be the most significant predictive factor of postoperative motor dysfunction, mIONM with TcMEP and Sp-SCEP was performed in our series instead of D-wave. D-wave could only evaluate the motor function and could not indicate the worsening side. While Sp-SCEP was stable in our series, it was not helpful to predict postoperative motor dysfunction.

In this study, deterioration of McCormick score at the final follow-up was significantly related to the length of peritumoral edema regardless of neither underlying disease, such as diabetes mellitus, nor the size or location of the tumors. Rajshekhar et al. conducted the prospective study and reported that preoperative neurological severity (Nurick grade), preoperative motor function, possible MEP monitoring were the most significant predictive factors for a favorable outcome at discharge (29). We could not show that Ratio MEP was significantly related to a favorable postoperative McCormick score probably because the number of patients in this study was quite small and we used McCormick score instead of Nurick grade. Some reported MEP monitoring could significantly improve McCormick's scores (21), others showed that MEP could increase the gross total resection rate but not affect postoperative McCormick score (12). It is still controversial whether MEP monitoring can significantly affect McCormick score at the final follow-up after spinal tumor surgery.

Park et al. examined the change of MEP on the deterioration of the motor function of 86 muscles in surgery of spinal ependymoma. When they used the cutoff ratio of 0.5, 97.4% of the muscles exhibiting postoperative weakness were recovered during the follow-up. They recommended all or none method of the cutoff value of zero in the evaluation of MEP (30). We performed a statistical analysis of the motor function of 46 muscles, Ratio MEP was the only significant negative factor predicting the deterioration at the final follow-up. In addition, ROC analysis revealed that Ratio MEP was significantly related to the deterioration at the final follow-up with the cutoff value of 0.23. By using this cutoff value, sensitivity, specificity, positive predictive value, and negative predictive value were 100, 88, 100, and 88%, respectively, which were quite high.

Muramoto et al. performed the same ROC analysis of 280 muscles in 37 patients who received surgery of intramedullary spinal tumor. This is the only study with a detailed ROC analysis to determine the cutoff value of MEP. Motor function of 51 muscles in 13 patients had deteriorated. Their estimated cutoff value of Ratio MEP was 0.12. Sensitivity, specificity, positive predictive value, and negative predictive value were 86, 74, 88, and 78%, which seemed lower than our study (31). While we used only one muscle at each extremity for MEP monitoring, they examined multiple muscles in each extremity. The difference in the results between the two studies might be caused by the difference in the way of MEP monitoring and of the number of muscles at one extremity. The cutoff value might be different depending on the institutions.

Milicevic examined the effect of IONM on the extent of tumor resection of 39 IMSCTs. Gross total resection was achieved in 89.7% but was not influenced by IONM (32). Cofano also reported that gross total resection was achieved in 84.3% of 249 IMSCTs and the use of IONM significantly affected the clinical condition at follow-up but not at discharge. However, the extent of resection was not associated with the use of IONM (33). van der Wal examined the effect of IONM for surgery of 78 EMSCTs. Total resection was achieved in 70.5% and mIONM with TcMEP and SEP yielded high to perfect sensitivity and high specificity for prediction of the deterioration of McCormick scores. van der Wal also mentioned that monitoring did not always determine the extent of resection because of surgeons' overruling of IONM. When the signal of IONM decreases, the surgeon temporarily stops resecting but usually proceeds resection with more caution or changes the dissecting plane (23). The resection strategy also depends on the nature of the tumor (23). In surgery for 500 IMSCTs, complete resection could be achieved in 77.2% of defined tumors and 41.7% of diffuse tumors even by the implementation of IONM with MEP (13).

In this study, we achieved gross total resection in 13 of 14 patients. In Case 2, MEP of the right upper and right lower extremities almost disappeared just after the dural incision. The tumor was tightly adhered to the spinal cord. In spite that Ratio MEP of this patient reached less than 0.23, we continued tumor resection to achieve subtotal resection. MEP did not recover until the end of the surgery. The patient showed left hemiparesis and hemihypesthesia but recovered to be able to walk and returned to work. However, the ratio of Sp-SCEP was also reduced to 0.33 in this case. If both of mIONM waned about postoperative deterioration, we might stop resection, especially in surgery of diffuse, malignant tumor, or tumor with severe adhesion.

## Conclusions

Ratio MEP was the most significant negative factor that predicts deterioration of motor weakness at spinal tumor surgery. The setting of the cutoff value should be more strict as compared to the brain surgery and might be different depending on the institutions. If both of mIONMs waned about postoperative deterioration, we might stop resection, especially in surgery of diffuse, malignant tumor, or tumor with severe adhesion.

## Data Availability Statement

The raw data supporting the conclusions of this article will be made available by the authors, without undue reservation.

## Ethics Statement

The studies involving human participants were reviewed and approved by 20200080. The patients/participants provided their written informed consent to participate in this study.

## Author Contributions

SY and KK: concept, design, and analysis and interpretation of data. HA: study supervision. KK: statistical analysis. TK: critically revising the article. SK, MI, TY, AK, MK, YS, HU, YT, and RH: acquisition of data. All authors contributed to the article and approved the submitted version.

## Funding

This work was supported by a Grant-in-Aid for Scientific Research by the Japan Society for the Promotion of Science (JSPS) (No. 20K09344).

## Conflict of Interest

The authors declare that the research was conducted in the absence of any commercial or financial relationships that could be construed as a potential conflict of interest.

## Publisher's Note

All claims expressed in this article are solely those of the authors and do not necessarily represent those of their affiliated organizations, or those of the publisher, the editors and the reviewers. Any product that may be evaluated in this article, or claim that may be made by its manufacturer, is not guaranteed or endorsed by the publisher.

## Supplementary Material

Supplementary Video 1 Cervical ependymoma without abnormal findings on MEP.

Supplementary Video 2 Tumor with neurenteric cyst with MEP signals suddenly disappearing during surgery.

## Abbreviations

IONM: Intraoperative neuromonitoring
IMSCT: Intramedullary spinal cord tumor
EMSCT: Extramedullary spinal cord tumor
MEP: Motor-evoked potential
SEP: Sensory-evoked potential
mIONM: Multimodal intraoperative monitoring
SCEP: Spinal cord evoked potential
TcMEP: Motor-evoked potential by transcranial stimulation.

## References

[1] Elsberg CA. Diagnosis and Treatment of Surgical Diseases of the Spinal Cord and Its Membranes. Philadelphia, W. B. Saunders Company (1916).

[2] Kopelson G Linggood RM Kleinman GM Doucette J Wang CC. Management of intramedullary spinal cord tumors. Radiology. (1980) 135:473–9. 10.1148/radiology.135.2.7367644 7367644

[3] Yasargil MG Antic J Laciga R de Preux J Fideler RW Boone SC. The microsurgical removal of intramedullary spinal hemangioblastomas. Report of twelve cases and a review of the literature. Surg Neurol. (1976) 1:141–8. 986698

[4] Nuwer MR Dawson EG Carlson LG Kanim LK Sherman JE. Somatosensory evoked potential spinal cord monitoring reduces neurologic deficits after scoliosis surgery: results of a large multicenter survey. Electroencephalogr Clin Neurophysiol. (1995) 96:6–11. 10.1016/0013-4694(94)00235-D 7530190

[5] Ginsburg HH Shetter AG Raudzens PA. Postoperative paraplegia with preserved intraoperative somatosensory evoked potentials. Case report. J Neurosurg. (1985) 63:296–300. 10.3171/jns.1985.63.2.0296 4020453

[6] Kearse LA Jr Lopez-Bresnahan M McPeck K Tambe V. Loss of somatosensory evoked potentials during intramedullary spinal cord surgery predicts postoperative neurologic deficits in motor function [corrected]. J Clin Anesth. (1993) 5:392–8. 10.1016/0952-8180(93)90103-L 8217175

[7] Kitagawa H Itoh T Takano H Takakuwa K Yamamoto N Yamada H. Motor evoked potential monitoring during upper cervical spine surgery. Spine. (1989) 10:1078–83. 10.1097/00007632-198910000-00009 2588056

[8] Dennis GC Dehkordi O Millis RM Cole AN Brown DS Paul OA. Monitoring of median nerve somatosensory evoked potentials during cervical spinal cord decompression. J Clin Neurophysiol. (1996) 13:51–9. 10.1097/00004691-199601000-00005 8988285

[9] Iwasaki H Tamaki T Yoshida M Ando M Yamada H Tsutsui S. Efficacy and limitations of current methods of intraoperative spinal cord monitoring. J Orthop Sci. (2003) 8:635–42. 10.1007/s00776-003-0693-z 14557928

[10] Morota N Deletis V Constantini S Kofler M Cohen H Epstein FJ. The role of motor evoked potentials during surgery for intramedullary spinal cord tumors. Neurosurgery. (1997) 41:1327–36. 10.1097/00006123-199712000-00017 9402584

[11] Quiñones-Hinojosa A Lyon R Zada G Lamborn KR Gupta N Parsa AT. Changes in transcranial motor evoked potentials during intramedullary spinal cord tumor resection correlate with postoperative motor function. Neurosurgery. (2005) 56:982–93. 15854246

[12] Choi I Hyun SJ Kang JK Rhim SC. Combined muscle motor and somatosensory evoked potentials for intramedullary spinal cord tumour surgery. Yonsei Med J. (2014) 55:1063–71. 10.3349/ymj.2014.55.4.1063 24954338 PMC4075368

[13] Westphal M Mende KC Eicker SO. Refining the treatment of spinal cord lesions: experience from 500 cases. Neurosurg Focus. (2021) 50:E22. 10.3171/2021.2.FOCUS201107 33932931

[14] Ruschel LG Aragão A de Oliveira MF Milano JB Neto MC Ramina R. Correlation of intraoperative neurophysiological parameters and outcomes in patients with intramedullary tumors. Asian J Neurosurg. (2021) 16:243–8. 10.4103/ajns.AJNS_234_20 34268146 PMC8244684

[15] Krammer MJ Wolf S Schul DB Gerstner W Lumenta CB. Significance of intraoperative motor function monitoring using transcranial electrical motor evoked potentials (MEP) in patients with spinal and cranial lesions near the motor pathways. Br J Neurosurg. (2009) 23:48–55. 10.1080/02688690802563349 19234909

[16] Tanaka S Tashiro T Gomi A Takanashi J Ujiie H. Sensitivity and specificity in transcranial motor-evoked potential monitoring during neurosurgical operations. Surg Neurol Int. (2011) 2:111. 10.4103/2152-7806.83731 21886884 PMC3162799

[17] Kurokawa R Kim P Itoki K Yamamoto S Shingo T Kawamoto T. False-positive and false-negative results of motor evoked potential monitoring during surgery for intramedullary spinal cord tumors. Oper Neurosurg (Hagerstown). (2018) 14:279–87. 10.1093/ons/opx113 29462450 PMC6057499

[18] Kimchi G Knoller N Korn A Eyal-Mazuz Y Sapir Y Peled A. Delayed variations in the diagnostic accuracy of intraoperative neuromonitoring in the resection of intramedullary spinal cord tumors. Neurosurg Focus. (2021) 50:E21. 10.3171/2021.2.FOCUS201084 33932929

[19] Hyun SJ Rhim SC. Combined motor and somatosensory evoked potential monitoring for intramedullary spinal cord tumor surgery: correlation of clinical and neurophysiological data in 17 consecutive procedures. Br J Neurosurg. (2009) 23:393–400. 10.1080/02688690902964744 19637010

[20] Chang SH Park YG Kim DH Yoon SY. Monitoring of motor and somatosensory evoked potentials during spine surgery: intraoperative changes and postoperative outcomes. Ann Rehabil Med. (2016) 40:470–80. 10.5535/arm.2016.40.3.470 27446784 PMC4951366

[21] Kang H Gwak HS Shin SH Woo MK Jeong IH Yoo H. Monitoring rate and predictability of intraoperative monitoring in patients with intradural extramedullary and epidural metastatic spinal tumors. Spinal Cord. (2017) 55:906–10. 10.1038/sc.2017.43 28485386

[22] Sala F Palandri G Basso E Lanteri P Deletis V Faccioli F. Motor evoked potential monitoring improves outcome after surgery for intramedullary spinal cord tumors: a historical control study. Neurosurgery. (2006) 58:1129–43. 10.1227/01.NEU.0000215948.97195.58 16723892

[23] van der Wal EC Klimek M Rijs K. Scheltens-de Boer M, Biesheuvel K, Harhangi BS: Intraoperative neuromonitoring in patients with intradural extramedullary spinal cord tumor: a single-center case series. World Neurosurg. (2021) 147:e516–23. 10.1016/j.wneu.2020.12.099 33383201

[24] Lee S Cho DC Rhim SC Lee BJ Hong SH Koo YS. Intraoperative monitoring for cauda equina tumors: surgical outcomes and neurophysiological data accrued over 10 years. Neurospine. (2021) 18:281–9. 10.14245/ns.2040660.330 34218610 PMC8255760

[25] Ando M Tamaki T Yoshida M Kawakami M Kubota S Nakagawa Y. Intraoperative spinal cord monitoring using combined motor and sensory evoked potentials recorded from the spinal cord during surgery for intramedullary spinal cord tumor. Clin Neurol Neurosurg. (2015) 133:18–23. 10.1016/j.clineuro.2015.03.004 25837236

[26] Koyanagi I Iwasaki Y Isu T Abe H Akino M Kuroda S. Spinal cord evoked potential monitoring after spinal cord stimulation during surgery of spinal cord tumors. Neurosurgery. (1993) 33:451–9. 10.1227/00006123-199309000-00015 8413877

[27] Duffau H Capelle L Sichez J. Direct spinal cord electrical stimulations during surgery of intramedullary tumoral and vascular lesions. Stereotact Funct Neurosurg. (1998) 4:180–9. 10.1159/000029662 10461104

[28] Cannizzaro D Mancarella C Nasi D Tropeano MP Anania CD Cataletti G. Intramedullary spinal cord tumors: the value of intraoperative neurophysiological monitoring in a series of 57 cases from two Italian centres. J Neurosurg Sci. (2019). 10.23736/S0390-5616.19.04758-1 31565906

[29] Rajshekhar V Velayutham P Joseph M Babu KS. Factors predicting the feasibility of monitoring lower-limb muscle motor evoked potentials in patients undergoing excision of spinal cord tumors. J Neurosurg Spine. (2011) 14:748–53. 10.3171/2011.1.SPINE10310 21438657

[30] Park JH Lee SH Kim ES Eoh W. Analysis of multimodal intraoperative monitoring during intramedullary spinal ependymoma surgery. World Neurosurg. (2018) 120:e169–80. 10.1016/j.wneu.2018.07.267 30096497

[31] Muramoto A Imagama S Ito Z Ando K Tauchi R Matsumoto T. The cutoff amplitude of transcranial motor evoked potentials for transient postoperative motor deficits in intramedullary spinal cord tumor surgery. Spine. (2014) 39:E1086–94. 10.1097/BRS.0000000000000421 24875959

[32] Milicevic M Solari D Illic R Frio F Stanimirovic A Savic D. The impact of intraoperative monitoring on extent of resection and long-term neurological outcomes: a series of 39 intramedullary ependimomas. Turk Neurosurg. (2020) 30:252–62. 10.5137/1019-5149.JTN.27471-19.2 32091124

[33] Cofano F Giambra C Costa P Zeppa P Bianconi A Mammi M. Management of extramedullary intradural spinal tumors: the impact of clinical status, intraoperative neurophysiological monitoring and surgical approach on outcomes in a 12-year double-center experience. Front Neurol. (2020) 11:598619. 10.3389/fneur.2020.598619 33391161 PMC7775672

